# No Evidence for a Role of Adipose Tissue-Derived Serum Amyloid A in the Development of Insulin Resistance or Obesity-Related Inflammation in hSAA1^+/−^ Transgenic Mice

**DOI:** 10.1371/journal.pone.0072204

**Published:** 2013-08-15

**Authors:** Sofie Ahlin, Maja Olsson, Bob Olsson, Per-Arne Svensson, Kajsa Sjöholm

**Affiliations:** 1 Department of Molecular and Clinical Medicine, Institute of Medicine, The Sahlgrenska Academy at the University of Gothenburg, Gothenburg, Sweden; 2 Department of Neurochemistry and Psychiatry, Institute of Neuroscience and Physiology, The Sahlgrenska Academy at the University of Gothenburg, Gothenburg, Sweden; Virginia Tech, United States of America

## Abstract

Obesity is associated with a low-grade inflammation including moderately increased serum levels of the acute phase protein serum amyloid A (SAA). In obesity, SAA is mainly produced from adipose tissue and serum levels of SAA are associated with insulin resistance. SAA has been described as a chemoattractant for inflammatory cells and adipose tissue from obese individuals contains increased numbers of macrophages. However, whether adipose tissue-derived SAA can have a direct impact on macrophage infiltration in adipose tissue or the development of insulin resistance is unknown. The aim of this study was to investigate the effects of adipose tissue-derived SAA1 on the development of insulin resistance and obesity-related inflammation. We have previously established a transgenic mouse model expressing human SAA1 in the adipose tissue. For this report, hSAA1^+/−^ transgenic mice and wild type mice were fed with a high fat diet or normal chow. Effects of hSAA1 on glucose metabolism were assessed using an oral glucose tolerance test. Real-time PCR was used to measure the mRNA levels of macrophage markers and genes related to insulin sensitivity in adipose tissue. Cytokines during inflammation were analyzed using a Proinflammatory 7-plex Assay. We found similar insulin and glucose levels in hSAA1 mice and wt controls during an oral glucose tolerance test and no decrease in mRNA levels of genes related to insulin sensitivity in adipose tissue in neither male nor female hSAA1 animals. Furthermore, serum levels of proinflammatory cytokines and mRNA levels of macrophage markers in adipose tissue were not increased in hSAA1 mice. Hence, in this model we find no evidence that adipose tissue-derived hSAA1 influences the development of insulin resistance or obesity-related inflammation.

## Introduction

Obesity is associated with adipose tissue inflammation and increased macrophage infiltration [1], [2]. In addition, obesity influences systemic responses to inflammation [3], [4] including moderately increased serum levels of the acute phase protein serum amyloid A (SAA) [5]. It has been suggested that these processes contribute to obesity-related metabolic disturbances such as insulin resistance and the development of type 2 diabetes [2], [4]–[6].

SAA has been extensively studied in many different aspects but its function is still unclear. SAA is an apolipoprotein and, in the circulation, it is mainly found associated with the HDL particle [7], [8]. It has been suggested that SAA is involved in processes such as cholesterol transport [9]–[12], lipolysis [5], [13], and opsonization [14], and may induce production of proinflammatory cytokines [5], [15]. In response to acute inflammation, SAA is produced by the liver [16], [17], and circulating levels of SAA can rise thousand-fold during the acute phase response [18], [19]. During non-acute phase, we [20] and others [21] have identified adipose tissue as the main source of SAA in obese subjects.

It is well established that obesity is tightly linked to insulin resistance [22], [23]. Several studies indicate that SAA may be a part of this link. Serum levels of SAA are associated with insulin resistance [24]–[26] and in vitro studies have shown that recombinant SAA can down regulate the expression of insulin signaling and glucose homeostasis related genes in adipocytes [27], [28]. It has also been suggested that that adipose tissue inflammation, including macrophage infiltration, is involved in the development of insulin resistance [1], [2]. SAA has been suggested to have chemoattractant properties [29]–[31] and may therefore increase macrophage infiltration. Hence, adipose tissue-derived SAA may have direct effects on the development of insulin resistance and type 2 diabetes.

We have previously reported the generation of a transgenic mouse model expressing human SAA1 (hSAA1) in the adipose tissue with moderately elevated circulating levels of SAA comparable those seen in human obesity [32]. In this study we used this model to investigate whether adipose tissue-derived hSAA1 plays a direct role in obesity-related inflammation and the development of insulin resistance.

## Materials and Methods

### Ethics Statement

All animal study protocols were approved by the local Ethics Committee for Animal Studies at the Administrative Court of Appeals in Gothenburg, Sweden (Permit numbers 281–2008 and 328–2009).

### Animals

The generation of hSAA1^+/−^ transgenic mice (hSAA1 mice) expressing human SAA1 in the adipose tissue under the control of the aP2 promoter has previously been reported [32]. The hSAA1 mice display moderately elevated circulating levels of SAA where SAA is found in the HDL-containing lipoprotein fraction. Hence the model is mimicking the native form of SAA in the human circulation where SAA is found associated with HDL. In the present experiment heterozygous hSAA1 mice (n = 37) and their wt littermates (n = 40) were weaned at 3 weeks of age and housed 3–7 per cage. The animals were kept in a temperature controlled (25°C) facility with a 12 hour dark-light cycle and were given ad libitum access to food and water. At the age of 10 weeks groups of wt and hSAA1 mice were matched according to sex, weight and body composition and were given either normal chow (NC) or a pelleted high fat diet (HFD) (60 kcal% fat; D12492, Research Diets, New Brunswick, NJ) for 12 weeks. At the end of experiment, animals were fasted for 4 hours, sacrificed under Isoflurane anesthesia (Baxter Kista Sweden) and blood was collected using heart puncture. Adipose tissue depots were dissected, weighed, snap frozen in liquid nitrogen and stored in −80°C until further analysis.

### Growth and Body Composition

Body weight was recorded weekly from 10 weeks of age and analysis of body composition was performed at 10 and 18 weeks of age. Body composition was assessed using dual energy X-ray absorptiometry (DEXA) (Lunar PIXImus II Densitometer, software version 2.10.041, GE Healthcare, Waukesha, WI), in animals anesthetized with Isoflurane.

### Oral Glucose Tolerance Test

At 21 weeks of age, the animals fed with HFD (n = 39) underwent an oral glucose tolerance test. In brief, animals were fasted for 4 hours and glucose solution (400 mg/ml, 2 g/kg) was administrated by oral gavage and blood was sampled from the tail vein. Samples were obtained before glucose administration and 15, 30, 60 and 120 minutes after. Levels of blood glucose and insulin were analyzed using the Accu-Check glucometer (Roche, Stockholm, Sweden) and an ultrasensitive mouse insulin enzyme-linked immunosorbent assay (ELISA) Kit (Chrystal Chem Inc., Downers Grove, IL), respectively.

### RNA Preparation and Gene Expression Analysis

Adipose tissue was homogenized using TissueLyser (Qiagen, Chatsworth, CA) and RNA was isolated using the RNeasy lipid tissue mini kit (Qiagen). Next, the High Capacity cDNA RT kit (Applied Biosystems, Foster City, CA) was used to generate cDNA from the RNA preparations and gene expression was then analyzed using real-time PCR. The reaction mixture contained TaqMan Master Mix, TaqMan Gene expression assays and cDNA corresponding to 10 ng RNA per reaction. The following TaqMan Gene expression assays were used for analyzing gene expression: rplp0 (Mm99999273_gh), SAA1/2 (Hs00761940_s1), Saa3 (Mm00441203_m1), Cd68 (Mm03047340_m1), Emr1 (Mm00802529_m1), Irs1 (Mm01278327_m1), Glut4 (Slc2a4) (Mm000436615_m1), Irs2 (Mm03038438_m1) and Adipoq (Mm00456425_m1). Amplification and detection of specific products was performed with the ABI PRISM 7900HT Sequence Detection System (Applied Biosystems) using default cycle parameters. A standard curve with serial dilution of cDNA synthesized from pooled RNA was used. All samples and standards were analyzed in triplicate.

### Plasma Analyses

Plasma was isolated by centrifugation (3000 g, 4°C, 10 minutes). Levels of human SAA and mouse SAA were analyzed using the human SAA ELISA kit (Biosource, Camarillo, CA) and the mouse SAA ELISA kit (Tridelta Development Ltd., Co, Kildare Ireland), respectively. Levels of IFN-γ, IL-10, IL-12p70, IL-1β, IL-6, TNF-α and CXCL-1 were assessed in male mice fed with HFD (n = 19) using Mouse Proinflammatory 7-plex Assay Ultra-sensitive Kit (Meso Scale Diagnostic, LLC, Gaithersburg) and analyzed on a SECTOR imager instrument with MSC Discovery Workbench analysis software (Meso Scale).

### Statistical Analysis

Data are reported as mean ± SEM unless otherwise stated. Statistical analyses were performed using PASW 19.0 (Chicago, IL). The Mann-Whitney U-test was used to investigate possible differences between groups. Repeated measures analysis of variance (ANOVA) was used to assess possible differences in growth between groups. A p-value of less than 0.05 was considered significant.

## Results

### SAA Expression, Growth and Body Composition

In line with previous analyses in male hSAA1 mice, we confirmed that hSAA1 is expressed in gonadal and retroperitoneal white adipose tissue and that serum levels of hSAA is increased after administration of HFD [32]. In this study we also found the same patterns in female hSAA1 mice (data not shown). In line with our previous data [32], mouse SAA adipose tissue expression and plasma levels were higher in HFD groups (data not shown). Furthermore, as shown previously [32] there was a significant reduction (females) or trend towards reduction (male) of mSAA3 expression in the gonadal adipose tissue in hSAA1 mice fed with HFD compared to wt mice on the same diet, indicating that hSAA is functional in the mouse.

**Figure 1:**
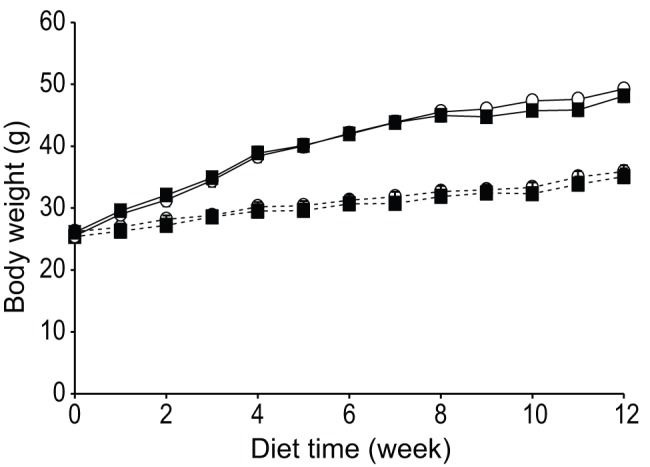
Animal growth curves. Growth curves for male hSAA1 mice (filled squares) and wt mice (open circles) fed either with a HFD (solid line) or NC (dashed line) for 12 weeks. n = 8–10 per group.

Furthermore, for HFD- or NC-fed groups, similar growth patterns in hSAA1 and wild type mice were observed throughout the experiment for both male (Figure 1) and female animals (Figure S1). In line with these results, hSAA1 and wild type mice displayed similar body composition at 18 w of age (data not shown).

### Glucose Tolerance and Adipose Tissue mRNA Levels of Genes Related to Insulin Sensitivity

**Figure 2:**
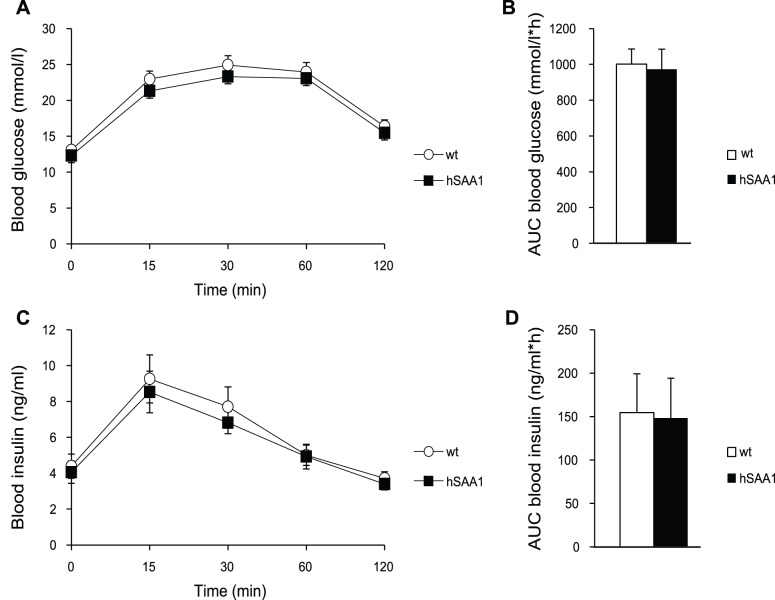
Levels of blood glucose and blood insulin during an oral glucose tolerance test. A) Blood glucose levels during an oral glucose tolerance test in male wt mice (open circles) and male hSAA1 mice (filled squares) fed with HFD. B) Blood glucose area under the curve (AUC) in male mice fed with HFD. C) Blood insulin levels during oral glucose tolerance test in male wt mice (open circles) and male hSAA1 mice (filled squares) fed with HFD. D) Blood insulin area under the curve (AUC) in male mice fed with HFD. n = 9–10 per group.

After 11 weeks of HFD administration, blood glucose and insulin levels were similar in male wt and hSAA1 mice (Figure 2a and 2c). No differences in blood glucose or insulin were seen at 0, 15, 30, 60 and 120 minutes after glucose administration (Figure 2a and c) and the area under the curve did not differ between wt and hSAA1 mice (Figure 2b and d). Female hSAA1 mice displayed no difference in blood glucose levels during the oral glucose tolerance test compared to their wt controls and there were no significant difference in blood glucose area under the curve (Figure S2a and S2b). Blood insulin levels in female hSAA1 mice were significantly lower 15 minutes after glucose administration compared to female wt mice. However, insulin area under the curve did not display any significant difference between female wt and hSAA1 mice (Figure S2c and S2d).

**Figure 3:**
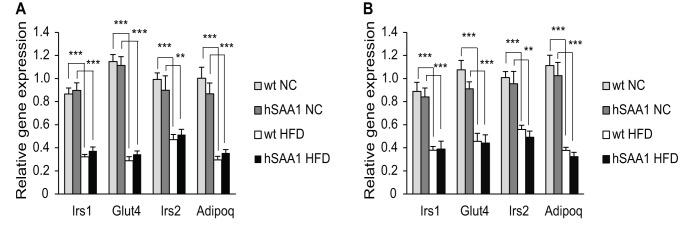
Adipose tissue mRNA levels of genes related to insulin sensitivity. mRNA levels of insulin sensitivity-related genes in (A) the gonadal fat depot and (B) the retroperitoneal fat depot in male mice. n = 8–10 per group. *indicates p<0.05, **p<0.01 and ***p<0.001.

Adipose tissue mRNA levels of genes related to insulin sensitivity, Irs1, Irs2, Glut 4 and adiponectin, were similar in male hSAA1 mice and their wt controls in both depots investigated (Figure 3a and b). Furthermore, expression of all four genes was significantly down-regulated in adipose tissue depots from male animals fed with HFD compared to those fed with NC. In female mice, gonadal and retroperitoneal adipose tissue expression levels were similar regardless of genotype except for Glut4 in the gonadal depot which displayed significantly lower levels of Glut4 in wt mice fed with HFD (Figure S3a and S3b). In the retroperitoneal fat depot, a majority of the genes related to insulin sensitivity displayed a significant reduction/or a trend towards down-regulation in the groups fed with HFD (Figure S3b).

### Levels of Inflammatory Markers in Adipose Tissue and Plasma

**Figure 4:**
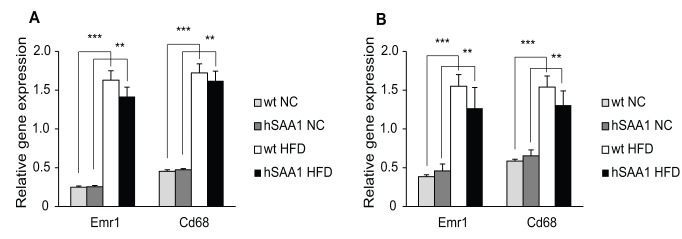
Adipose tissue mRNA levels of macrophage markers. mRNA levels of macrophage markers in (A) the gonadal fat depot and (B) the retroperitoneal fat depot in male mice. n = 8–10 per group. *indicates p<0.05, **p<0.01 and ***p<0.001.

Both wt and hSAA1 males and females had significantly higher mRNA levels of macrophage markers, Cd68 and Emr1, in both the gonadal and retroperitoneal adipose tissue when fed with HFD compared with NC (Figure 4a and b; Figure S4a and 4Sb). However, there were no significant differences in mRNA levels of Cd68 and Emr1 between hSAA1 mice and their wt littermates.

Levels of inflammatory markers were measured in plasma from HFD-fed male mice. With the exception of lower levels of CXCL1 in hSAA1 mice, no significant differences in IFN-γ, IL-10, IL-12p70 or IL-1β or TNF-α between hSAA1 and wt mice were found (Table 1).

**Table 1:** Inflammatory markers in plasma.

	Wtn = 10	hSAA1n = 9
IFN-γ	1.6±0.38	2.6±0.9
IL-10	38.1±4.1	56.2±14.9
IL-12 p70	78.1±11.7	131.4±41.0
IL-1β	4.3±0.7	3.0±0.5
IL-6	27.5±2.4	35.2±8.2
TNF-α	1.9±0.2	1.7±0.2
CXCL1	464.8±44.5*	345.8±29.7*

Levels of inflammatory markers in plasma (pg/ml) in male mice fed with HFD. n = 9–10 per group.

* indicates p<0.05.

## Discussion

In this study we find no evidence that adipose tissue-derived human SAA1 influences insulin sensitivity or obesity-related inflammation in mice. We here demonstrate that hSAA1 transgenic mice have similar glucose and insulin responses as their wt controls during an oral glucose tolerance test. Furthermore, mRNA levels of genes related to insulin sensitivity in adipose tissue from hSAA1 mice were not decreased compared to those seen in wt animals. In this study we also demonstrate that mRNA levels of macrophage markers in adipose tissue and circulating levels of pro-inflammatory markers are not increased in hSAA1 mice. This indicates that adipose tissue-derived human SAA1 does not have proinflammatory properties and does not affect obesity-related inflammation or insulin sensitivity in mice.

In our study we have used a hSAA1 transgenic mouse model which mimics the human obese state, i.e. expression of hSAA1 in the adipose tissue and moderately elevated circulating levels of SAA associated to its’ natural carrier HDL [32]. Effects of SAA on lipoprotein profiles and atherosclerosis have previously been studied *in vivo* using viral over-expression and genetically modified mice producing SAA in the liver [33]–[35]. However, we believe that our model is better suited when studying the long-term effects of adipose tissue-derived SAA1 on metabolic or inflammatory function *in vivo*.

We show in this study that oral glucose tolerance and mRNA levels of genes related to insulin sensitivity are not decreased in hSAA1 mice. This was an unexpected finding as previous studies show that serum levels of SAA are associated with diabetes and insulin resistance in both humans and mice [27], [36], [37]. Furthermore, in type 2 diabetes patients, PPAR-γ agonists reduce the serum levels of SAA in parallel with an improvement of glycemic status and insulin sensitivity [38]. In addition, *in vitro* studies indicate that SAA may down-regulate the expression of insulin signaling and glucose homeostasis related genes [13], [27], [28]. However, in our study, glucose response during an oral glucose tolerance test was similar in hSAA1 mice and wt controls in spite of hSAA1 mice displaying circulating levels of hSAA comparable to serum levels of SAA in obese humans. In line with these results, we found no decrease in the expression of genes related to insulin sensitivity in hSAA1 mice. Hence, our results from this mouse model indicate that hSAA1 may be an inert marker of insulin resistance instead of an active player in the development of insulin resistance.

The mRNA levels of the macrophage markers Cd68 and Emr1 in adipose tissue were unchanged in hSAA1 mice suggesting that adipose tissue-derived hSAA1 does not function as a local chemoattractant for inflammatory cells. Previous studies using recombinant SAA have shown that SAA can act as a chemoattractant for neutrophils and monocytes, and the chemoattractant responsiveness for SAA is higher in type 2 diabetes patients [29], [30]. Another suggested mechanism for the chemoattractant effect of SAA is induction of Ccl2 [27], a factor known to induce macrophage migration in adipose tissue [6]. However, our results suggest that adipose tissue-derived hSAA1 does not influence to the local adipose tissue inflammation seen in obesity.

Obesity is not only associated with a local inflammation in adipose tissue but also with low-grade inflammation [3], [4]. This is illustrated by an increase in circulating levels of SAA but also other cytokines such as IL-6 and TNF-α [39], [40]. Recombinant SAA can induce cytokine production in monocytes both at the RNA and protein level [15], [30]. To investigate whether hSAA1 has proinflammatory systemic effects we measured inflammatory markers in plasma in animals fed with HFD. With the exception of the lower levels of CXCL1 in hSAA1 mice, no significant differences in IFN-γ, IL-10, IL-12p70 or IL-1β or TNF-α between hSAA1 and wt mice were found. Hence, we found no sign of increased cytokines in the circulation of hSAA1 mice.

Research devoted to the study of SAA function has been going on for more than 30 years. This research suggests that SAA is a multifunctional molecule that may affect a large number of processes in the body [5], [9]–[12], [14], [15]. Recently, the choice of methods for investigating SAA function has been criticized regarding whether previous experiments actually imitate the physiological condition [41]. A majority of the previous studies have been performed using a de-lipidated, recombinant protein that is not identical to endogenous human SAA [28]–[30]. The physiological relevance of this recombinant protein has been questioned since it was recently reported to display different properties compared to endogenous hSAA [42]–[44]. This indicates that some of the published data regarding the function of SAA need to be reevaluated.

In conclusion, results from our hSAA1 mouse model, mimicking the human obese state with increased expression of hSAA1 in adipose tissue and moderately elevated circulating levels of SAA, imply that adipose tissue-derived hSAA1 does not influence insulin sensitivity or obesity-related inflammation in mice. This opens up the possibility that the moderately elevated serum level of SAA in obesity could be an inert marker of insulin resistance instead of playing an active role in the development of type 2 diabetes and the obesity-related inflammation.

## Supporting Information

Figure S1 **Animal growth curves.** Growth curves for female hSAA1 mice (filled squares) and wt mice (open circles) fed either with a HFD (solid line) or a NC (dashed line) for 12 weeks. n = 10 per group. Data are presented as mean ± SEM.
(EPS)

Figure S2 **Levels of blood glucose and blood insulin during an oral glucose tolerance test.** A) Blood glucose levels during an oral glucose tolerance test in female wt mice (open circles) and hSAA1 mice (filled squares) fed with HFD. B) Blood glucose area under curve (AUC) in female mice fed with HFD. C) Blood insulin levels during an oral glucose tolerance test in female wt mice (open circles) and hSAA1 mice (filled squares) fed with HFD. D) Blood insulin area under curve (AUC) in female mice fed with HFD. n = 10 per group. *indicates p<0.05, **p<0.01. Data are presented as mean ± SEM.
(EPS)

Figure S3 **Adipose tissue mRNA levels of genes related to insulin sensitivity.** mRNA levels of insulin signaling related genes in (A) gonadal fat depots and (B) retroperitoneal fat depots in female mice. n = 10 per group. *indicates p<0.05, **p<0.01. Data are presented as mean ± SEM.
(EPS)

Figure S4 **Adipose tissue mRNA levels of macrophage markers.** mRNA levels of macrophage markers in (A) gonadal fat depots and (B) the retroperitoneal fat depot in female mice. n = 10 per group. *indicates p<0.05, **p<0.01. Data are presented as mean ± SEM.
(EPS)
